# Effects of 6-week high-intensity interval training and moderate-intensity continuous training on classroom attention in children: a randomized controlled study

**DOI:** 10.3389/fphys.2026.1876163

**Published:** 2026-07-03

**Authors:** Yijie Ma, Yaqin Xu, Rangxi Jin, Dexin Wang, Chao Chen, Na Jiang, Xinbo Zhang

**Affiliations:** 1 School of Athletic Performance, Shanghai University of Sport, Shanghai, China; 2 School of Physical Education, Shanghai University of Sport, Shanghai, China; 3 College of Physical Education, Dalian University, Dalian, Liaoning, China; 4 Medicine and Sports Health Promotion Medical College, Dalian University, Dalian, Liaoning, China

**Keywords:** chronic effect, classroom attention, HIIT, physical activity, school

## Abstract

**Introduction:**

Although physical activity has been shown to enhance children’s cognitive and attentional functions, the comparative effects of High-Intensity Interval Training (HIIT) and Moderate-Intensity Continuous Training (MICT) on classroom attention behaviors remain unclear. This study compared the effects of 6-week HIIT and MICT interventions on children’s classroom attention behaviors using AI-based behavior capture in an ecological environment.

**Methods:**

Participants were randomly assigned to HIIT, MICT, or control groups, with interventions integrated into physical education lessons for six weeks. An Intel RealSense multimodal system monitored 760 minutes of core subject classes before and after the intervention. Key attentional behavior markers, including head posture, eye-gaze, blinking, and face detection, were analyzed.

**Results:**

Following the 6-week intervention, both exercise groups demonstrated significantly higher overall attentional behavior scores compared to the control group. Inter-group comparisons revealed that the HIIT group significantly outperformed the MICT group in head posture stability, face detection persistence, and overall attention levels (p < 0.05). No significant differences were observed between the two exercise groups regarding eye-gaze consistency and blinking frequency (p > 0.05).

**Discussion:**

These findings indicate that integrating HIIT or MICT into physical education effectively enhances classroom attention behaviors. However, HIIT provides a greater advantage in maintaining stable physical orientation toward instructional areas of interest. Educators should consider both exercise intensity and movement characteristics when designing physical education activities aimed at promoting classroom attentional engagement.

## Introduction

In the contemporary primary education system, classroom attention is regarded as a pivotal predictor of children’s academic achievement and long-term cognitive development (Welsh et al., 2010; Anzeneder et al., 2023). Particularly during the learning of core subjects such as Chinese, Mathematics, and English, the ability to maintain sustained attention directly modulates the efficiency of knowledge acquisition and the depth of cognitive processing (Donnelly et al., 2016; Liu et al., 2024). However, prevailing sedentary learning patterns and increasingly heavy cognitive loads have led to widespread challenges of attentional lapse among students (Budde et al., 2008; Pellegrini and Davis, 1993). To date, numerous studies have identified physical activity (PA) as an effective non-pharmacological intervention for enhancing the mental health and cognitive functions of children and adolescents (García-Hermoso et al., 2021; Ludyga et al., 2020). According to the systematic review by Donnelly et al. (2016), a significant positive correlation exists between PA, physical fitness, and academic achievement, suggesting that regular exercise can effectively improve executive function and attentional performance in children.

While the general benefits of PA are well-established, evidence suggests that the efficacy of exercise interventions is significantly moderated by various factors (García-Hermoso et al., 2021). A meta-analysis by Ludyga et al. (2020) demonstrated that the impact of exercise on children’s cognitive function depends on the duration, frequency, and specific modality of the intervention. Specifically, long-term exercise programs exert a unique cumulative effect on cognitive improvement, whereas the complexity of movements and the intensity distribution are likely key determinants of the variance in cognitive gains.

Regarding the selection of exercise modalities, the “Cognitive Engagement Hypothesis” postulates that the cognitive benefits of exercise depend not only on physiological intensity but also on the level of cognitive challenge inherent in the activity (Tomporowski et al., 2008). Gallotta et al. (2012) noted that exercise modalities involving coordinative challenges yield more pronounced improvements in children’s attention compared to traditional physical education, implying that complex movement sequences can more effectively activate higher-order cognitive centers. Furthermore, research by Anzeneder et al. (2023) emphasized the synergistic effect between the depth of cognitive challenge and exercise duration. High-Intensity Interval Training (HIIT), which combines explosive movements with high metabolic demand, aligns well with this theoretical framework. Compared to Moderate-Intensity Continuous Training (MICT) characterized by repetitive movements, HIIT not only triggers the release of neurotransmitters but also provides sustained cognitive stimulation through complex rhythmic patterns (Taubert et al., 2015).

Much of prior research on PA and children’s attention relies on standardized cognitive assessments, such as the d2 Test of Attention (Brickenkamp et al., 2010), in which participants must rapidly identify target stimuli while inhibiting responses to visually similar distractors. These measures offer strong validity and enable comparisons across studies. However, they may not fully capture the dynamic and context-dependent nature of attention as it unfolds in everyday environments (Kingstone et al., 2008). Accordingly, future research should examine whether findings derived from well-validated laboratory tasks generalize to real-world settings. In authentic educational contexts, attention extends beyond reaction times to discrete stimuli; rather, it emerges as a coordinated pattern of behaviors, including gaze, posture, and task engagement (Birmingham et al., 2009; Zaletelj and Košir, 2017). For instance, an individual’s ability to maintain postural discipline (head posture stability; Raca et al., 2015; Zhong et al., 2023), the locking of visual focus on instructional targets (eye-gaze direction), and the level of active engagement toward the teaching area (persistence of facial-feature detection) are all dimensions that provide more intuitive and ecologically valid assessments of classroom attentional engagement (Stiefelhagen, 2002; Birmingham et al., 2008).

To objectively capture these multidimensional behavioral features, the present study employed an automated multimodal assessment system based on 3D depth-sensing technology (Intel RealSense) to conduct continuous monitoring of actual classroom teaching. We hypothesized that following the 6-week intervention, the HIIT group would demonstrate significantly superior overall attention scores compared to both the MICT and control groups. This hypothesis is grounded in the premise that the explosive and coordinative movements inherent in HIIT more effectively stimulate the proprioceptive system and elevate neural arousal levels, thereby fostering enhanced behavioral regulation in the classroom.

## Methods

### Participants and design

This was a randomized intervention study with three groups and two distinct measurement occasions. An *a priori* calculation with G*Power (Faul et al., 2007) for a 3 × 2 (Group × Phase) analysis of variance (ANOVA) with repeated measurements, based on a middle effect size (*f* = 0.25), power of 0.95, and the alpha level of.05, resulted in a minimal sample size of 66 persons. Participants were 72 students (24 women and 48 men) recruited from a primary school in China. Inclusion criteria were the target age (10–11 years), a normal BMI, normal intellectual capacity (as confirmed by their teachers), no physical limitations and sufficient physical fitness to participate in physical education. The study was approved by the ethics committee of the first author’s institution. The guardians of all enrolled children provided written informed consent prior to the experiment.

### Procedure

The study comprised three distinct phases: a pretest, a 6-week exercise intervention phase, and a posttest. The pretest took place one week before the exercise intervention. Participants self-reported their age and gender, and completed physical fitness assessment. Furthermore, participants’ classroom attention was assessed during all core subject classes (Chinese, Math, and English) that week, which were 19 classes lasting 40 minutes each (i.e., 760 min of observation). The exercise intervention lasted for six consecutive weeks, with three 28-min training sessions per week (i.e., 18 sessions in total). Each session consisted of a 6-min warm-up, the specific physical intervention (high-intensity, moderate-intensity, or curriculum-based physical education; PE; 18 min), and a cool-down at the end (4 min). All training sessions were delivered within the regular PE lessons and led by qualified PE teachers. In the posttest, one week after completing the exercise intervention, participants’ classroom attention was measured for the second time; each participant’s attention scores were derived from a total of 760 minutes of observation during the core subject classes.

### Intervention

**Figure 1:**
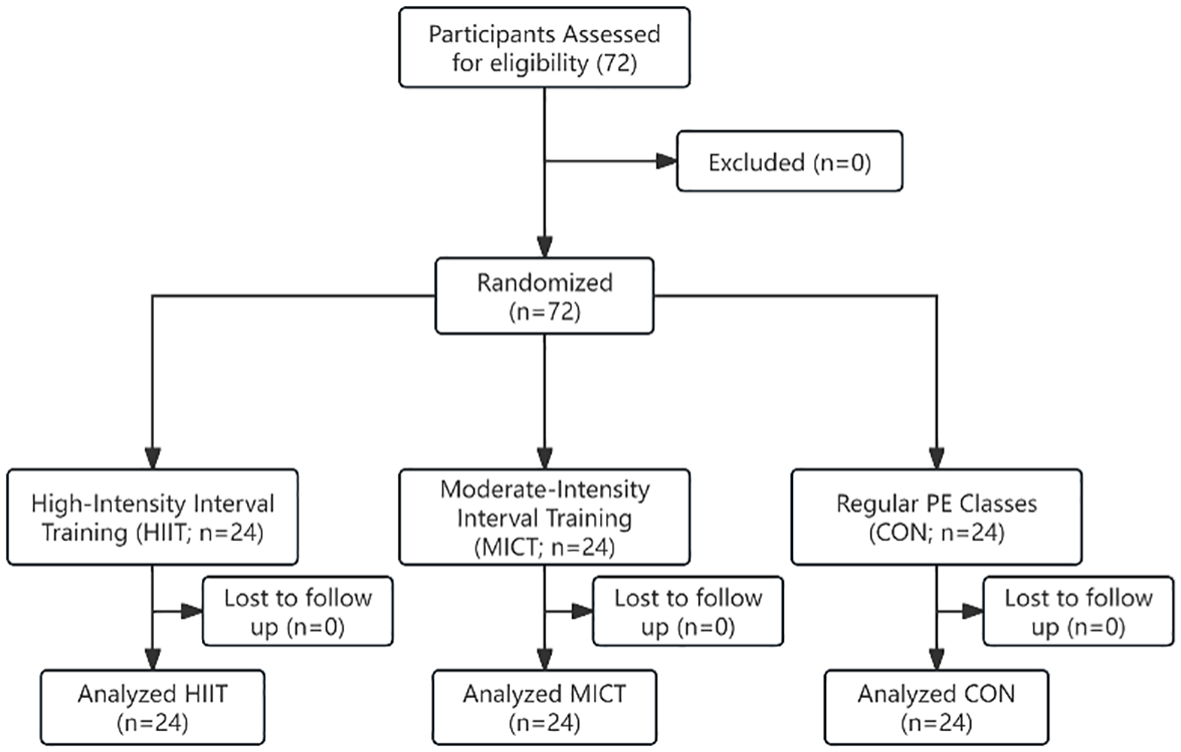
Flow chart showing the experimental design of the study.

We used stratified randomization to assign participants to one of two exercise intervention groups (high-intensity, moderate-intensity) or to a conventional, curriculum-based PE group (**Figure 1**). Participants were first grouped into strata according to their gender (male, female). Within the stratum, participants were then assigned to one of the three study groups by using randomization function in Microsoft Excel (Microsoft, Redmond, WA). The exercise intervention groups completed the same 6-min warm-up (jogging, dynamic stretching) and 4-min cool-down (stretching, relaxation) at the start and the end of each session, respectively. The specific physical intervention (high-intensity, moderate-intensity) took place in-between and lasted for 18 min.

Participants in the *high-intensity exercise group* completed a high-intensity interval training (HIIT) protocol. The HIIT included four exercises in a circuit (jumping jacks, cross-jumps, vertical jumps, and burpees), each performed for 30 seconds. The protocol followed a 1:1 work-to-rest ratio: participants started with jumping jacks and cross-jumps for a total of 60-sec work, followed by 60-sec rest, then continued with vertical jumps and burpees for another 60-sec work, and finished with 60-sec rest. Participants completed the circuit four times. The intensity for the work intervals was set at ≥85% of HRmax (Mezcua-Hidalgo et al., 2019), which corresponds to ≥170 bpm in children aged 10–11 years (Gelbart et al., 2017). During the rest intervals, participants remained stationary without performing any active movement. Participants in the *moderate-intensity exercise group* performed a moderate-intensity continuous training (MICT). The training consisted of continuous aerobic running, including activities such as lap running, curve running, and shuttle runs. The activity was continuous, with no rest intervals during the training protocol. The intensity was set at 60–69% of HRmax, which corresponds to 120–138 bpm in children aged 10–11 years (Gelbart et al., 2017). Participants in the *conventional, curriculum-based PE group* did not receive any specific training intervention and served as a control group. They attended conventional physical education classes that included activities such as school-wide calisthenics and ball games. A strict requirement for this group was that the content must not utilize the specific training modalities employed by the two intervention groups. Activities that could potentially confound the research outcomes—such as long-distance running, sprinting, or short-duration continuous jumping—were avoided during the course of the study.

### Measures

#### Training intensity

To ensure compliance with the prescribed training intensity, participants’ heart rates were continuously monitored using a Polar chest-strap heart rate device (Polar Electro, Kempele, Finnland). For each session in both intervention groups (HIIT and MICT), five participants per group were randomly selected for heart rate monitoring in each session to evaluate group-level compliance with the prescribed intensity ranges.

#### Body mass index

Participants’ weight (kg) and height (cm) were obtained with participants dressed in sports clothing and barefoot. Weight was measured using a calibrated digital scale with 0.1 kg precision, and height was measured with a stadiometer accurate to 0.5 cm. Body mass index (BMI) was computed as weight divided by height squared (kg/m²).

#### Physical fitness

Physical fitness was assessed using the National Student Physical Fitness Assessment Standards (Revised 2014), a standardized protocol widely used in China to evaluate the physical fitness of school-aged children and adolescents (Ministry of Education of the People’s Republic of China, 2014). The test measures five major components of physical fitness (speed, flexibility, coordination, strength, and endurance) and vital capacity. Speed was assessed with a 50-m sprint, in which participants covered a distance of 50 m from a standing position in as short a time (in seconds) as possible. Flexibility was measured with the sit-and-reach test, in which participants reached forward from a seated, legs-extended position. The maximal reach distance (in centimeters) was recorded, with higher values indicating greater flexibility. Coordination was measured with the 1-min rope-skipping test, in which participants performed as many correct skips as possible in 60 seconds. Strength was assessed with the 1-min sit-up test, in which participants performed as many correctly executed sit-ups as possible in 60 seconds. Endurance was tested with the 50 m × 8 shuttle run, in which participants ran back and forth over a 50-m course eight times, and the total completion time was recorded. Finally, vital capacity was assessed with a spirometer. Participants took a maximal inhalation followed by a complete exhalation, and the volume of air exhaled (in milliliters) was recorded.

#### Classroom attention

Participants’ attention behaviors were assessed with an Attention Analysis and Video Capture System (Intel RealSense Online Classroom Attention Assessment System; Ningyu Technology Co., Shanghai). The system uses a RealSense 3D depth-sensing camera installed in the classroom to collect real-time, multidimensional behavioral data. Computer-vision algorithms analyze three-dimensional head-position parameters, eye-gaze vectors, eyelid periodicity (open/closed ratio), and facial-feature stability to determine the spatial relationship between participants’ behavioral orientation and the instructional interface (Zhong et al., 2023). The system integrated four behavioral metrics associated with classroom attentional engagement: head posture stability, eye-gaze consistency, blinking frequency, and face detection persistence.

Based on the Intel RealSense framework, this study evaluated classroom attention across four dimensions. Head posture, eye-gaze consistency, and face detection share a unified computational logic: their raw scores are defined as the temporal proportion (f = $\sum t_{i\;}$/T) spent within predefined Areas of Interest (AOIs), such as the teacher or the blackboard, where T represents the effective tracking duration and it represents the time oriented toward the AOI.

Specifically, the system identifies attentional deviations through established behavioral thresholds: head posture is derived from Euler angles (yaw, pitch, and roll), where a yaw deviation >30° lasting ≥ 3s is categorized as an off-task event (Zhong et al., 2023). Eye-gaze consistency detects “pseudo-attention” by identifying gaze shifts ≥ 200 pixels for a duration ≥ 5s. Face detection serves both as a prerequisite for landmark tracking and as a direct indicator of physical orientation toward the instructional source (Zaletelj and Košir, 2017). Meanwhile, blinking frequency status monitors arousal levels, with the raw score calculated as f = 1 - t/D (where t is the closure duration and D is the effective tracking duration); a single closure exceeding 2 s is utilized as the threshold for an inattention state (Kim et al., 2023).

To ensure data integrity, segments with facial occlusion >30% or landmark confidence <0.7 were automatically excluded. Excluded segments were treated as missing data and were removed from both the numerator and denominator when calculating the behavioral indices. For the final assessment, the four normalized metrics (x_1_ to x_4_) were integrated into an overall attention index (S) using a geometric mean formula, with equal weighting assigned to each dimension: S = $\sqrt[{4}]{x_{1}\times x_{2}\times x_{3}\times x_{4}}$. The geometric mean approach was selected because it reduces the influence of extreme values across individual dimensions and emphasizes balanced performance across multiple behavioral indicators, thereby minimizing the likelihood that any single indicator exerts undue influence on the overall composite score.

### Statistical analysis

To test the homogeneity of groups, separate one-way ANOVAs were conducted on participants’ age, BMI, physical fitness, and pretest attentional scores. To check the intensity of exercise intervention, means and standard deviations of participants’ heart rates were calculated. To test the study hypothesis, separate 3 (Group) × 2 (Phase) repeated measures ANOVAs were conducted on classroom attention scores to test whether attention changed from pretest to posttest among the study groups. In case of significant interaction, simple effects analyses were performed with Bonferroni adjustment. All analyses were performed with SPSS 29.0 (IBM Corp.; Armonk, NY, United States). The significance level was set at *p* <.05 (two tailed). Effect sizes were estimated using partial eta squared (ηp²), with values of 0.01–0.05 indicating small effects, 0.06–0.13 indicating medium effects, and values ≥ 0.14 indicating large effects.

## Results

**Figure 2:**
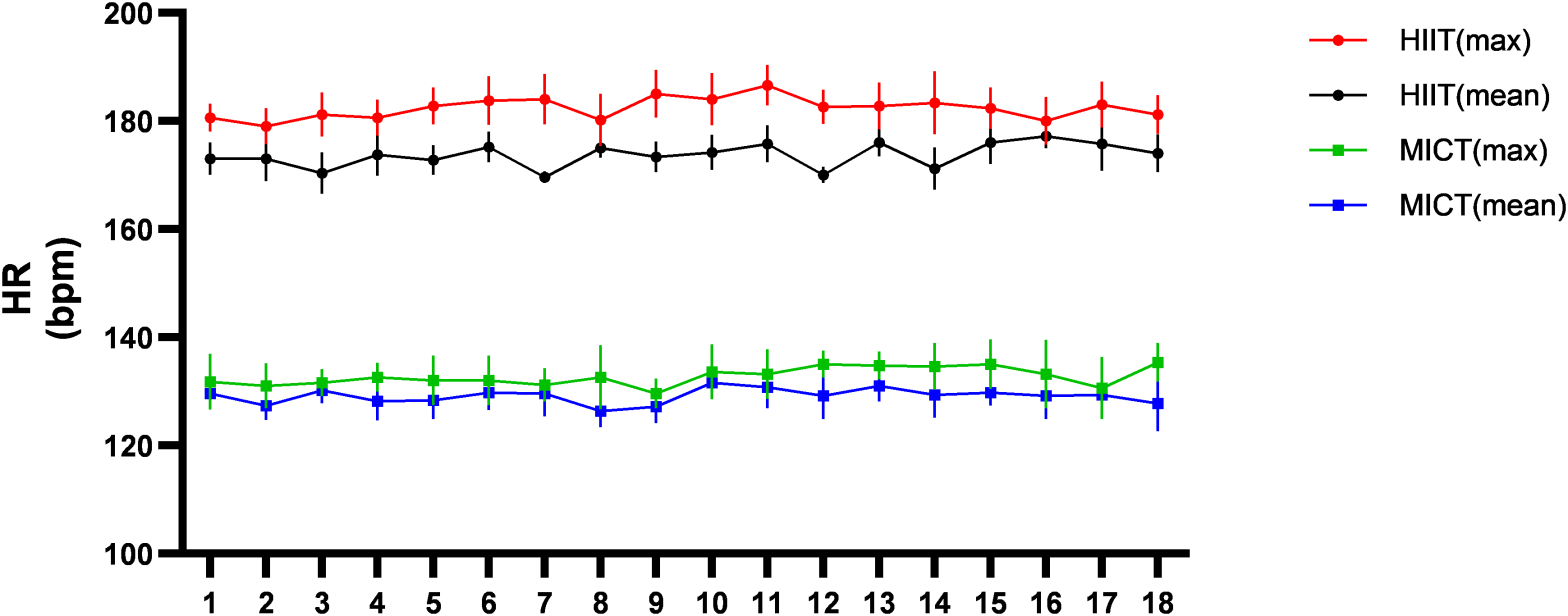
Heart rates in the exercise intervention groups during the training sessions.

Characteristics of the study groups are shown in **Table 1**. The groups did not differ in age, BMI, and physical fitness. The intensity of the exercise interventions was appropriate; the mean heart rates recorded during training sessions ranged from 173 to 182 bpm in the HIIT group and from 129 to 132 bpm in the MICT group (**Figure 2**).

**Table 1:** Characteristics of the study groups (means ± SDs).

Variables	HIIT (*n* = 24)	MICT (*n* = 24)	Conventional (*n* = 24)	*F*	*p*	η^2^
Age (years)	10.50 ± 0.51	10.38 ± 0.50	10.63 ± 0.50	1.50	.23	0.04
BMI	17.71 ± 1.72	17.78 ± 1.96	18.58 ± 1.40	1.91	.16	0.05
50-meter sprint (s)	9.03 ± 0.44	9.10 ± 0.36	9.26 ± 0.54	1.65	.20	0.05
Sit-and-reach (cm) (M)	9.18 ± 1.88	9.33 ± 1.44	8.53 ± 2.41	0.75	.48	0.02
1-min rope skipping (times)	99.63 ± 12.88	101.67 ± 13.42	95.63 ± 23.70	1.15	.32	0.03
1-min sit-ups (times)	35.50 ± 6.32	35.83 ± 5.55	34.08 ± 4.99	0.65	.53	0.02
50 m × 8 shuttle run (s)	108.17 ± 10.64	113.89 ± 8.16	112.13 ± 8.97	1.66	.20	0.05
Vital capacity (mL)	2192.08 ± 231.31	2191.67 ± 197.63	2166.67 ± 389.44	0.14	.87	0.01

Groups are stratified according to gender; each study group includes eight women and 16 men. HIIT, high-intensity interval training group; MICT, moderate-intensity continuous training group; Conventional, curriculum-based physical education group.

Classroom attention scores of the intervention and control groups are displayed in **Table 2**. All five scores (head posture stability, eye-gaze consistency, blinking frequency, face detection persistence, and overall attention level) improved from pretest to posttest, as indicated by the main effects of Phase. Moreover, the interaction effects were significant, indicating that the improvement in classroom attention varied across groups. Subsequent simple effects analyses revealed no between-group differences at pretest (*p*s >.70), whereas significant group differences emerged at posttest. In particular, participants in both intervention groups outperformed control participants in all five attention scores (*p*s <.001). Within the exercise intervention conditions, participants in the HIIT group demonstrated higher scores than those in the MICT group for head posture stability (*p* <.001), face detection persistence (*p* = .034), and overall attention level (*p* <.001), whereas the groups did not differ in eye-gaze consistency or blinking frequency (*p*s >.088).

**Table 2:** Means (± SDs) and ANOVA statistics for attentional variables.

Variables	HIIT	MICT	Conventional	ANOVA			
	(*n* = 24)	(*n* = 24)	(*n* = 24)	Effect	*F*	*p*	ηp^2^
Head posture stability				P	71.55	.001	0.51
Pre	0.60 ± 0.05	0.61 ± 0.06	0.61 ± 0.08	G	12.11	.001	0.26
Post	0.74 ± 0.03	0.66 ± 0.05	0.60 ± 0.06	P × G	37.85	.001	0.52
Eye-gaze consistency				P	55.53	.001	0.45
Pre	0.56 ± 0.09	0.53 ± 0.11	0.52 ± 0.11	G	10.62	.001	0.24
Post	0.71 ± 0.05	0.65 ± 0.13	0.52 ± 0.10	P × G	14.92	.001	0.30
Blinking frequency				P	13.25	.001	0.16
Pre	0.66 ± 0.08	0.67 ± 0.10	0.66 ± 0.09	G	5.97	.004	0.15
Post	0.76 ± 0.02	0.72 ± 0.08	0.64 ± 0.09	P × G	9.22	.001	0.21
Face detection persistence				P	39.07	.001	0.36
Pre	0.55 ± 0.09	0.56 ± 0.10	0.55 ± 0.11	G	7.99	.001	0.19
Post	0.71 ± 0.07	0.65 ± 0.10	0.54 ± 0.09	P × G	16.86	.001	0.33
Overall attention				P	116.88	.001	0.63
Pre	0.59 ± 0.06	0.59 ± 0.07	0.58 ± 0.08	G	13.34	.001	0.28
Post	0.73 ± 0.03	0.67 ± 0.07	0.57 ± 0.07	P × G	47.44	.001	0.58

P, Phase; G, Group; P × G, Phase × Group interaction; HIIT, high-intensity interval training group; MICT, moderate-intensity continuous training group; Conventional, curriculum-based physical education group.

## Discussion

The present study, through a 6-week randomized controlled intervention, found that physical exercise can significantly improve the attentional performance of primary school children in authentic classroom environments. This result supports the general conclusion by Donnelly et al. (2016) regarding exercise-promoting cognition. Crucially, although both HIIT and MICT protocols proved effective, the HIIT group showed significant statistical superiority across key metrics, including head posture, face detection, and overall attention indices. This elevation in scores may reflect an increased duration of active behavioral engagement, evidenced by students maintaining more stable frontal body orientations and more consistent visual orientation toward the predefined AOIs.

HIIT demonstrated greater improvements than MICT in overall classroom attentional behaviors and specific behavioral indicators, such as head posture stability and face detection persistence. These differences may be partially explained by the combined influence of higher physiological demands and greater movement complexity inherent in the HIIT protocol (Sabaghi et al., 2025; Poon et al., 2023; Buchheit and Laursen, 2013). From a physiological perspective, the high-intensity characteristics of HIIT (≥170 bpm) may induce greater levels of physiological arousal (Kemppainen et al., 2005; Sabaghi et al., 2025). Previous literature has suggested that higher-intensity exercise may be associated with increased catecholamine release (e.g., dopamine and norepinephrine) and enhanced neurotrophic responses, such as increased brain-derived neurotrophic factor levels (Alves et al., 2014; Taubert et al., 2015). These mechanisms have been hypothesized to contribute to enhanced arousal regulation and attentional readiness. Compared with continuous running activities in the MICT condition, the HIIT protocol involved more varied movement patterns and greater coordination demands, which may have increased cognitive participation during exercise sessions. Therefore, the observed benefits may reflect the combined effects of exercise intensity, movement complexity, and behavioral engagement rather than intensity alone (Gallotta et al., 2012; Mao et al., 2024).

Furthermore, the Cognitive Engagement Hypothesis may provide one possible explanation for the advantages of HIIT in classroom attentional behaviors. The HIIT protocol in this study included complex circuit movements such as burpees, vertical jumps, and power bounds, which not only imposed higher physiological demands but also required greater proprioceptive processing and movement coordination. Previous studies have suggested that exercise modes involving higher cognitive and coordinative demands may place greater demands on executive processes during task execution (Gallotta et al., 2012; Anzeneder et al., 2023). Specifically, improvements in head posture stability may indicate more stable behavioral orientation toward instructional targets (Raca et al., 2015), whereas improvements in face detection persistence may reflect greater maintenance of physical orientation toward instructional sources (Birmingham et al., 2008). This interpretation is consistent with the view that exercise modes involving greater coordinative and cognitive demands may promote stronger behavioral engagement during subsequent classroom activities.

Despite HIIT’s superiority in overall attentional control, the two exercise interventions showed similar improvement effects in eye-gaze consistency and blinking frequency, two behavioral indicators associated with visual orientation, with no significant differences between groups (ps >.05). This suggests that gaze-related behaviors and postural orientation behaviors may reflect partially distinct behavioral processes. Specifically, head posture stability and face detection persistence primarily reflect students’ physical orientation toward instructional targets, whereas eye-gaze consistency captures the stability of visual focus within those targets. Therefore, improvements in physical orientation do not necessarily translate into proportional improvements in gaze stability (Zaletelj and Košir, 2017). Eye-gaze consistency reflects the stability of behavioral orientation toward instructional targets and may be influenced by multiple factors related to visual orientation and attentional engagement (Taubert et al., 2015; Anzeneder et al., 2023). Previous studies have suggested that moderate-intensity exercise may already be sufficient to induce physiological responses associated with behavioral indicators of visual orientation (Taubert et al., 2015). Therefore, although HIIT provided higher physiological demands, the absence of group differences may indicate that both interventions were sufficient to elicit improvements in gaze-related behaviors, resulting in a possible ceiling effect within this behavioral domain (McMorris and Hale, 2012; Chang et al., 2012).

Furthermore, the non-significant difference in blinking frequency may reflect similarities in behavioral arousal responses between the two exercise interventions (Siegle et al., 2008). Blinking frequency is commonly regarded as a behavioral indicator associated with fatigue and visual load (Siegle et al., 2008). Since the intervention in this study was conducted within regular PE lessons and the testing environment was an authentic classroom lasting 760 minutes, both exercise modalities may have produced comparable physiological responses associated with maintaining classroom alertness. This suggests that exercise interventions that interrupt sedentary behavior and induce at least moderate physiological arousal (Lambrick et al., 2016), may be sufficient to support behavioral alertness without requiring extremely high exercise intensities (Anzeneder et al., 2023). This unbalanced pattern of improvement further highlights the multidimensional nature of classroom attentional behaviors. Although HIIT appeared to confer greater benefits in behavioral indicators related to physical orientation toward instructional targets, these findings should not be interpreted as direct evidence of deeper cognitive engagement or enhanced information processing. Students may maintain appropriate body posture and facial orientation toward instructional targets while simultaneously experiencing mind wandering or reduced cognitive engagement. Therefore, the present findings suggest that different components of classroom attention may respond differently to exercise interventions and should be considered separately when evaluating attentional outcomes (Chang et al., 2015).

Although the computer-vision system provided ecologically valid measurements of classroom attentional behaviors, the assessed indicators should be interpreted as behavioral proxies of classroom attentional engagement rather than direct measures of cognitive attention. In addition, the composite attention index was constructed as an equally weighted geometric mean of four behavioral indicators. Although this approach provides a useful summary measure of classroom attentional behaviors, the relative contribution of each indicator to attentional engagement has not been empirically established. Therefore, the overall attention index should be considered an exploratory summary measure rather than a validated composite, and conclusions based on this index should be interpreted with appropriate caution. Future research should examine the latent structure of these indicators using factor analytic and other validation approaches and should also explore alternative weighting schemes. Furthermore, the HIIT and MICT protocols differed not only in exercise intensity but also in movement complexity and coordinative demands. In addition, participants in the control group engaged in regular physical education activities, including calisthenics and ball games, while exercise intensity was not systematically monitored. Therefore, the independent effects of exercise intensity, cognitive-motor complexity, and exercise dose could not be fully separated in the present study. Finally, heart rate monitoring was conducted in only a small random subsample of participants during each intervention session. Although different participants were randomly selected across sessions, individual compliance with the prescribed exercise intensity could not be verified for all participants. Consequently, some uncertainty remains regarding intervention fidelity at the individual level.

## Conclusion

The 6-week HIIT and MICT interventions were both effective in improving the classroom attention of school-aged children. However, HIIT demonstrated significantly greater advantages in enhancing students’ overall attention scores, postural stability, and active engagement performance. Therefore, we suggest that educators, when designing school physical activity intervention programs, should fully consider the organic integration of exercise intensity and movement complexity to maximize the promotional effects of physical activity on children’s cognitive development.

## Data Availability

The raw data supporting the conclusions of this article will be made available by the authors, without undue reservation.

## References

[B1] Alves C. R. R. Tessaro V. H. Teixeira L. A. C. Murakava K. Roschel H. Gualano B. . (2014). Influence of acute high-intensity aerobic interval exercise bout on selective attention and short-term memory tasks. Perceptual Motor Skills 118, 63–72. doi: 10.2466/22.06.PMS.118k10w4 24724513

[B2] Anzeneder S. Zehnder C. Schmid J. Martin‐Niedecken A. L. Schmidt M. Benzing V. (2023). Dose–response relation between the duration of a cognitively challenging bout of physical exercise and children’s cognition. Scandinavian J. Med. Sci. Sports 33, 1439–1451. doi: 10.1111/sms.14370 37088931

[B3] Birmingham E. Bischof W. F. Kingstone A. (2008). Social attention and real-world scenes: The roles of action, competition and social content. Q. J. Exp. Psychol. 61, 986–998. doi: 10.1080/17470210701410375 18938281

[B4] Birmingham E. Bischof W. F. Kingstone A. (2009). Get real! Resolving the debate about equivalent social stimuli. Visual Cognition 17 (6–7), 904–924. doi: 10.1080/13506280902758044

[B5] Brickenkamp R. Schmidt-Atzert L. Liepmann D. (2010). d2-R: Test d2 – Revision: Aufmerksamkeits- und Konzentrationstest. (Göttingen, Germany: Hogrefe).

[B6] Buchheit M. Laursen P. B. (2013). High-intensity interval training, solutions to the programming puzzle. Sports Med. 43, 313–338. doi: 10.1007/s40279-013-0029-x 23539308

[B7] Budde H. Voelcker-Rehage C. Pietraßyk-Kendziorra S. Ribeiro P. Tidow G. (2008). Acute coordinative exercise improves attentional performance in adolescents. Neurosci. Lett. 441, 219–223. doi: 10.1016/j.neulet.2008.06.024 18602754

[B8] Chang Y.-K. Chu C.-H. Wang C.-C. Wang Y.-C. Song T.-F. Tsai C.-L. . (2015). Dose–response relation between exercise duration and cognition. Med. Sci. Sports Exercise 47, 159–165. doi: 10.1249/MSS.0000000000000383 24870572

[B9] Chang Y. K. Labban J. D. Gapin J. I. Etnier J. L. (2012). The effects of acute exercise on cognitive performance: A meta-analysis. Brain Res. 1453, 87–101. doi: 10.1016/j.brainres.2012.02.068 22480735

[B10] Donnelly J. E. Hillman C. H. Castelli D. Etnier J. L. Lee S. Tomporowski P. . (2016). Physical activity, fitness, cognitive function, and academic achievement in children: A systematic review. Med. Sci. Sports Exercise 48, 1197–1222. doi: 10.1249/MSS.0000000000000901 27182986 PMC4874515

[B11] Faul F. Erdfelder E. Lang A.-G. Buchner A. (2007). G*Power 3: A flexible statistical power analysis program for the social, behavioral, and biomedical sciences. Behav. Res. Methods 39, 175–191. doi: 10.3758/BF03193146 17695343

[B12] Gallotta M. C. Guidetti L. Franciosi E. Emerenziani G. P. Bonavolontà V. Baldari C. (2012). Effects of varying type of exertion on children’s attention capacity. Med. Sci. Sports Exercise 44, 550–555. doi: 10.1249/MSS.0b013e3182305552 21814148

[B13] García-Hermoso A. Ramírez-Vélez R. Lubans D. R. Izquierdo M. (2021). Effects of physical education interventions on cognition and academic performance outcomes in children and adolescents: A systematic review and meta-analysis. Br. J. Sports Med. 55, 1224–1232. doi: 10.1136/bjsports-2021-104112 34187782

[B14] Gelbart M. Ziv-Baran T. Williams C. A. Yarom Y. Dubnov-Raz G. (2017). Prediction of maximal heart rate in children and adolescents. Clin. J. Sport Med. 27, 139–144. doi: 10.1097/JSM.0000000000000315 27177205

[B15] Kemppainen J. Aalto S. Fujimoto T. Kalliokoski K. K. Långsjö J. Oikonen V. . (2005). High intensity exercise decreases global brain glucose uptake in humans. J. Physiol. 568, 323–332. doi: 10.1113/jphysiol.2005.091355 16037089 PMC1474763

[B16] Kim D. Park H. Kim T. Kim W. Paik J. (2023). Real-time driver monitoring system with facial landmark-based eye closure detection and head pose recognition. Sci. Rep. 13, 18264–18264. doi: 10.1038/s41598-023-44955-1 37880264 PMC10600215

[B17] Kingstone A. Smilek D. Eastwood J. D. (2008). Cognitive Ethology: A new approach for studying human cognition. British Journal of Psychology 99 (3), 317–340. doi: 10.1348/000712607X251243 17977481

[B18] Lambrick D. Stoner L. Grigg R. Faulkner J. (2016). Effects of continuous and intermittent exercise on executive function in children aged 8–10 years. Psychophysiology 53, 1335–1342. doi: 10.1111/psyp.12688 27314635

[B19] Liu Y. Dong X. He Q. Jia Y. (2024). Effects of acute rope skipping exercises of different exercise modes on cognitive function in 9–10-year-old children. Sci. Rep. 14, 29172–29172. doi: 10.1038/s41598-024-80987-x 39587327 PMC11589861

[B20] Ludyga S. Gerber M. Pühse U. Looser V. N. Kamijo K. (2020). Systematic review and meta-analysis investigating moderators of long-term effects of exercise on cognition in healthy individuals. Nat. Hum. Behav. 4, 603–612. doi: 10.1038/s41562-020-0851-8 32231280

[B21] Mao F. Huang F. Zhao S. Fang Q. (2024). Effects of cognitively engaging physical activity interventions on executive function in children and adolescents: A systematic review and meta-analysis. Front. Psychol. 15. doi: 10.3389/fpsyg.2024.1454447 39246315 PMC11377322

[B22] McMorris T. Hale B. J. (2012). Differential effects of differing intensities of acute exercise on speed and accuracy of cognition: A meta-analytical investigation. Brain Cogn. 80, 338–351. doi: 10.1016/j.bandc.2012.09.001 23064033

[B23] Mezcua-Hidalgo A. Ruiz-Ariza A. Suárez-Manzano S. Martínez-López E. J. (2019). 48-hour effects of monitored cooperative high-intensity interval training on adolescent cognitive functioning. Perceptual Motor Skills 126, 202–222. doi: 10.1177/0031512518825197 30665339

[B24] Ministry of Education of the People’s Republic of China (2014). National Student Physical Health Standard (Revised 2014). (Beijing, China: Ministry of Education of the People’s Republic of China.

[B25] Pellegrini A. D. Davis P. D. (1993). Relations between children’s playground and classroom behaviour. Br. J. Educ. Psychol. 63, 88–95. doi: 10.1111/j.2044-8279.1993.tb01043.x 8466835

[B26] Poon E. T.-C. Wongpipit W. Sun F. Tse A. C.-Y. Sit C. H.-P. (2023). High-intensity interval training in children and adolescents with special educational needs: A systematic review and narrative synthesis. Int. J. Behav. Nutr. Phys. Act. 20, 13–13. doi: 10.1186/s12966-023-01421-5 36759853 PMC9909882

[B27] Raca M. Kidzinski L. Dillenbourg P. (2015). Translating head motion into attention—towards processing of student's body-language. In: Proceedings of the 8th International Conference on Educational Data Mining (EDM 2015). 320–326.

[B28] Sabaghi A. Ebrahimi B. Yousofvand N. Hoseini R. (2025). Comparative effects of moderate-intensity continuous training and high-intensity interval training on ADHD symptoms and behavioral inhibition in children. Eur. J. Pediatr. 184, 183–183. doi: 10.1007/s00431-025-06022-x 39920369

[B29] Siegle G. J. Ichikawa N. Steinhauer S. (2008). Blink before and after you think: Blinks occur prior to and following cognitive load indexed by pupillary responses. Psychophysiology 45 (5), 679–687. doi: 10.1111/j.1469-8986.2008.00681.x 18665867

[B30] Stiefelhagen R. (2002). Tracking focus of attention in meetings. Proc. Fourth IEEE Int. Conf. Multimodal Interfaces, 273–280. doi: 10.1109/ICMI.2002.1167006 25079929

[B31] Taubert M. Villringer A. Lehmann N. (2015). Endurance exercise as an “endogenous” neuro-enhancement strategy to facilitate motor learning. Front. Hum. Neurosci. 9. doi: 10.3389/fnhum.2015.00692 26834602 PMC4714627

[B32] Tomporowski P. D. Davis C. L. Miller P. H. Naglieri J. A. (2008). Exercise and children’s intelligence, cognition, and academic achievement. Educ. Psychol. Rev. 20, 111–131. doi: 10.1007/s10648-007-9057-0 19777141 PMC2748863

[B33] Welsh J. A. Nix R. L. Blair C. Bierman K. L. Nelson K. E. (2010). The development of cognitive skills and gains in academic school readiness for children from low-income families. J. Educ. Psychol. 102, 43–53. doi: 10.1037/a0016738 20411025 PMC2856933

[B34] Zaletelj J. Košir A. (2017). Predicting students’ attention in the classroom from Kinect facial and body features. EURASIP J. Image Video Process. 2017, 80–80. doi: 10.1186/s13640-017-0228-8 38164791

[B35] Zhong R. He L. Wang H. Yuan L. Li K. Liu Z. (2023). Attention-guided Huber loss for head pose estimation based on improved capsule network. Entropy 25 (7), 1024. doi: 10.3390/e25071024 37509971 PMC10378512

